# Use of Coercive Measures during Involuntary Psychiatric Admission and Treatment Outcomes: Data from a Prospective Study across 10 European Countries

**DOI:** 10.1371/journal.pone.0168720

**Published:** 2016-12-29

**Authors:** Paul McLaughlin, Domenico Giacco, Stefan Priebe

**Affiliations:** 1 Unit for Social & Community Psychiatry, WHO Collaborating Centre for Mental Health Services Development, East London NHS Foundation Trust and Queen Mary University of London, United Kingdom; 2 Wolfson Institute, Queen Mary University of London, United Kingdom; SPAIN

## Abstract

To assess the association between different types of coercive measures (forced medication, seclusion, and restraint) used during involuntary psychiatric admission and two treatment outcomes: retrospective views of patients towards their admission and length of inpatient stay. A secondary analysis was conducted of data previously gathered by the EUNOMIA study (n = 2030 involuntarily detained inpatients across 10 European countries, of whom 770 were subject to one or more coercive measures). Associations between coercive measures and outcomes were tested through multivariable regression models adjusted for patients' socio-demographic and clinical characteristics. Use of forced medication was associated with patients being significantly less likely to justify their admission when interviewed after three months. All coercive measures were associated with patients staying longer in hospital. When the influence of other variables was considered in a multi-variate analysis, seclusion remained as a significant predictor of longer inpatient stay, adding about 25 days to the average admission. Of the three coercive measures, forced medication appears to be unique in its significant impact on patient disapproval of treatment. While all coercive measures are associated with patients staying longer in hospital, only use of seclusion is associated with longer inpatient stays independently of coerced patients’ having higher symptom scores at the time of admission.

## Introduction

The use of coercion in mental health care remains common practice in jurisdictions across the world. As well as involuntary admission to hospital under statutory powers of detention, the most obvious forms of coercive practice are those referred to as ‘coercive measures’–forced administration of psychotropic medication against the patient’s will, involuntary confinement of the patient in isolation or seclusion, and manual or mechanical restraint of the patient’s limbs or body to prevent free movement. Despite the widespread use of coercive measures, however, there is a remarkable lack of empirical evidence as to their association with treatment outcomes [1, 2]. A recent review of the literature [3] found only 13 studies on the relationship between coercive measures and treatment outcomes, and reported mixed findings, with the quality of all the studies rated as poor.

Given their widespread use [4], the association between coercive measures and treatment outcomes is clearly important. Quite apart from the physical risks that go with the use of force, qualitative studies consistently show that coercive measures can be experienced by patients as humiliating and distressing [5, 6], and consideration has started to be made of the psychological risks of their use [7, 8]. On mainly humanitarian grounds, many countries have put strategic plans in place to reduce or eliminate the use of coercive measures, particularly the use of seclusion and restraint, but evidence as to their association with outcomes should also inform and influence these important organisational and cultural changes.

The aims of this study were to answer the following research questions:

1. Is there an association between the use of coercive measures and the views of patients as to whether their involuntary admission to hospital was right or wrong?
2. Is the use of coercive measures associated with patients having shorter or longer stays in hospital?

## Methods

To answer these questions a secondary analysis was conducted of data collected by a large European cohort study.

### Sample

The EUNOMIA study (European Evaluation of Coercion in Psychiatry and Harmonisation of Best Clinical Practice) was a multicentre prospective cohort study that gathered data from a large sample of involuntarily detained patients recruited across 12 countries. The main aim of the study was to assess cross-national variations in the use of coercion, their influencing factors, and their outcomes. It screened for eligibility and recruited involuntarily detained patients in one to five centres across 12 countries between July 2003 and December 2005. All patients included in the study gave informed consent, and national and local ethics approval was obtained in each participating country. A full description of study design and methodology is provided by the study authors [9]. For this secondary analysis, data were included from 10 countries: Bulgaria, the Czech Republic, Greece, Germany, Italy, Lithuania, Poland, Spain, Sweden and the United Kingdom. Israel and the Slovak Republic also participated in the EUNOMIA study but data from these countries were not included due to inadequacies in their dataset.

### Procedures and measures

This analysis included data collected for: age, gender, ICD-10 diagnosis [10], length of index admission, and assessment of symptoms using the 24-item Brief Psychiatric Rating Scale (BPRS) [11], administered in most cases within the first three days after admission and very rarely up to seven days after admission. Also included in the analysis were data gathered on the following measures:

Coercive measures–Clinical records of each use of coercive measures during the first four weeks of admission (or less, if the patient was discharged earlier) using the following definitions: *seclusion*, the involuntary confinement of a patient in a locked room alone, which may be designed specifically for this purpose; *restraint*, fixing at least one of the patient’s limbs with a mechanical device or being held by one or more member of staff for longer than 15 minutes; and *forced medication*, using manual restraint or strong psychological pressure (involving at least three members of staff) to administer, orally or by injection, medication against the patient’s will.

Retrospective views on admission–Participants were interviewed by one of the researchers and asked to rate at three months, irrespective of whether they were still in hospital or not, whether they thought their involuntary admission to hospital (or their subsequent detention within seven days after admission for those admitted voluntarily) was justified. This was rated on a Likert-type scale ranging from 0 (‘entirely wrong’) to 10 (‘entirely right’). For the analysis the ratings were dichotomised as less than, equal to, or greater than the neutral middle point of 5, so that participants were classified into those with neutral or negative views and those with positive views.

### Statistical analysis

Descriptive statistics were calculated for: a) number and percentages of patients who were subject to coercive measures–forced medication, seclusion, and restraint; b) number and percentages of patients who felt after three months that their index admission was justified; c) number and percentages or mean and standard deviations for length of index admission and the other socio-demographic and clinical variables collected, as appropriate.

Explanatory variables for retrospective views on admission at three months and length of index admission were investigated using two multivariable regression models. Models assessing the association of each type of coercive measure with the dichotomised variable reflecting retrospective justification were analysed using logistic regression. Models assessing the relationship between each type of coercive measure and length of index admission were analysed using linear regression. Variables that were significant in univariable models at p = 0.1 were then included in multivariable models. The final models were adjusted for BPRS scores at baseline and countries’ effect inserted as dummy variables. An alpha level of 0.05 was used for all statistical tests. All analyses were conducted using IBM SPSS for Statistics version 22.

## Results

A total of 2030 involuntary patients were included in the study admitted across the 10 countries. Of those, 770 (37.9%) were subject to one or more coercive measures in the first four weeks of their admission or less, if they were discharged sooner, resulting in 1462 incidents of their use. There were significant differences between countries in the frequency of use of coercive measures per patient, and in the frequency of use of each type of coercive measure per country. There were no significant differences between patients who experienced coercive measures and those who did not for gender, age, employment, and living situation. There were, however, significant differences between the two groups for diagnosis of schizophrenia and BPRS score at baseline, with patients who experienced coercive measures being significantly more likely to have a diagnosis of schizophrenia (68% versus 60%, p = .004) and significantly more likely to have a higher BPRS score at baseline (58 versus 52, p = < .001). A full description of the use of coercive measures by country and by type, and participating patients’ socio-demographic and clinical characteristics, is provided elsewhere [12].

### Retrospective views of patients

1353 patients (66.7%) were interviewed at three months and, of those, 152 (11.2%) were still in hospital when interviewed. Of the responses, 847 (62.6%) approved of their admission and 506 (37.4%) disapproved. In the univariable analysis forced medication was the only coercive measure that was significantly associated with patients being less likely to justify their involuntary treatment. Of the 1353 patients interviewed at three months, 556 (41.09%) were subject to forced medication, and of those 57.7% justified their admission, compared with 64.6% of those not subject to forced medication. In the multivariable analysis the significant association for forced medication remained (p = .004), and treatment disapproval was significantly associated with female patients, and patients with a diagnosis of schizophrenia or other psychotic disorder. Table 1 shows the associations between independent and dependent variables in the analysis.

**Table 1:** Logistic regression analysis of association between coercive measures (and other variables) and retrospective justification of index admission after 3 months.

	Univariable analysis			Multivariable analysis		
	Odds ratio	CI	p	Odds ratio	CI	p
Forced medication	.746	.587 - .947	.016	.684	.528 - .886	.004
Seclusion	1.105	.625–1.953	.732	-	-	-
Restraint	.832	.741–1.273	.971	-	-	-
Gender	1.560	1.249–1.947	< .001	1.550	1.224–1.961	< .001
Age	.998	.988–1.008	.699	-	-	-
Past hospitalisation	1.066	.829–1.369	.620	-	-	-
Diagnosis, schizophrenia	.874	.693–1.103	.256	.698	.506 - .962	.029
Diagnosis, mood disorders	1.399	1.041–1.879	.026	-	-	-
Diagnosis, other	.880	.658–1.178	.390	.614	.415 - .909	.015
BPRS at baseline	1.064	.902–1.254	.463	-	-	-

### Length of inpatient stay

Data on length of index admission were available for 1913 patients (94%). The 556 patients subject to forced medication had a mean length of stay of 46.6 (55.1) days, compared to 38.2 (47.4) days for those not subject to forced medication, forced medication adding just under 9 days to the average length of stay, which was statistically significant (p = .001). A total of 84 patients were subject to seclusion, with a mean length of stay of 64.8 (92.3) days, compared to 39.5 (46.7) days for those not subject to seclusion, seclusion adding about 25 days to the average length of stay, which was statistically significant (p = < .001). A total of 439 patients were subject to restraint, with a mean length of stay of 44.6 (47.4) days, compared to 39.4 (57) days for those not subject to restraint, restraint adding about 5 days to the average length of stay, which was just short of statistical significance (p = .057). In the univariable analysis forced medication and seclusion were both significantly associated with patients staying longer in hospital, with restraint showing a trend towards the same, just short of statistical significance. Significant associations were also found for patients with a previous inpatient admission, patients with a diagnosis of schizophrenia or other psychotic disorder, patients with a diagnosis of an affective disorder, and patients with a high BPRS at baseline. In the multivariable analysis the significant association for forced medication did not remain, but significant associations remained for seclusion (p = < .001), for patients with a diagnosis of an affective disorder, and patients with a high BPRS at baseline. Table 2 shows the associations between independent and dependent variables in the analysis.

**Table 2:** Linear regression analysis of association between coercive measures (and other variables) and length of index admission.

	Univariable analysis			Multivariable analysis		
	B	CI	p	B	CI	p
Forced medication	8.349	3.437–13.262	.001	.023	-2.855–7.966	.354
Seclusion	25.241	14.383–36.100	< .001	.106	15.329–36.978	< .001
Restraint	5.156	-.159–10.471	.057	-	-	-
Gender	.062	-3.852–3.976	.975	-	-	-
Age	.066	-.133 –.265	.514	-	-	-
Past hospitalisation	9.881	-14.894 –-4.867	< .001	.036	-.752–8.736	.099
Diagnosis, schizophrenia	16.802	11.620–20.785	< .001	-	-	-
Diagnosis, mood disorders	-7.598	-13.447 –-1.750	.011	-.066	-14.357 –-2.911	.003
Diagnosis, other	-17.253	-22.900 –-11.607	< .001	-.125	-21.638 –-10.185	< .001
BPRS at baseline	7.505	4.048–10.962	< .001	.071	1.716–9.467	.005

## Discussion

### Main findings

Of the three coercive measures, forced medication was the only one significantly associated with patient disapproval of treatment. This finding was derived from patients being interviewed three months after their index admission, suggesting that the negative impact of forced medication was still felt by patients several months after the experience of forced medication had taken place. Although all three types of coercive measures were associated with patients staying longer in hospital, only use of seclusion was found to be associated with patients staying significantly longer in hospital, independently of coerced patients having higher BPRS scores at the time of admission.

### Strengths and limitations of the study

This study analysed data gathered as part of the largest international study of coercive measures to date. The data used in this analysis were gathered across 10 European countries over a period of a year and a half. Inclusion criteria and definition of coercive measures were uniform across the 10 countries. The large sample size (n = 2030) is a major strength of this study, allowing for good statistical power to enable data analysis and interpretation. Clearly the differing legal and health systems existing across the 10 countries, and the differing standards of implementation and training around the use of coercive measures, will have had an effect on the data–but country effects were controlled for in the multivariable analyses.

Most prospective studies have some attrition over the study period and EUNOMIA was not unusual in this regard. Data on retrospective views were only available for about two thirds of participants in the study, with about one third missing. Study centres were based at between one and five hospitals in one city in each country (two cities in Spain), and only about 50% of patients in each centre were eligible for the study and, of those, about 48% consented to participate. All of these factors may have affected the representativeness of the data and potentially created some selection bias.

### Comparison of findings with previous literature

Psychiatric forced medication is a remarkably under-studied practice [13]. This study supports the findings of other smaller studies that use of forced medication is associated with patient disapproval of treatment, much more so than seclusion or restraint [14, 15]. This was also a conclusion reached by a multi-site study in England, which described forced medication as ‘toxic’ in its impact on patient attitudes towards treatment in general [16]. A German study that interviewed 90 patients, however, found that mechanical restraint was perceived as ‘more profound’ in its impact on patient satisfaction than involuntary medication [17]. Although some have suggested that there may be cultural and national preferences in attitudes towards coercive measures [18], forced medication was found to be significantly associated with patient disapproval across all 10 countries in the EUNOMIA study, which would suggest that the impact of national and cultural differences may be limited.

Other studies, using a much smaller data-set, have found that coercive measures, and seclusion in particular, are associated with patients staying longer in hospital [19–23]. This might be explained, of course, by coerced patients being simply more unwell and taking longer to recover, and EUNOMIA did find that coerced patients were significantly more likely to have a higher BPRS score at baseline (58 versus 52, p = < .001). A higher BPRS score at baseline was one of the factors controlled for in the multivariable analysis, however, and yet the significant association for seclusion remained, meaning that secluded patients being more unwell does not fully explain the association.

The results of this study confirm the findings of other studies that the negative impact of coercion on patients’ views may be greater with patients with a diagnosis of schizophrenia [24, 25] and with female patients [26, 27]. The reasons for these findings are unclear. It is possible that patients with a diagnosis of schizophrenia may have a more fragile ‘sense of self’ that might make them more vulnerable to the psychological impact of coercion [28], and statistically women are more likely than men to report stress reactions to potentially traumatic events [29]. It is not unusual on psychiatric wards for coercive measures to be carried out by teams of predominantly male staff, although the effectiveness of all-male teams has been questioned [30], and this may be one of the reasons that the negative impact on female patients appears to be greater.

### Implications of this study

The impact of forced medication on patient disapproval of treatment is significant even after three months. The negative impact of forced medication, and treatment dissatisfaction more generally, may be greater with female patients and patients with a diagnosis of schizophrenia. More research is needed to explore the reasons why treatment disapproval is higher in these groups. Services should take these findings into account when offering debrief and support to patients.

Use of seclusion is associated with patients staying longer in hospital, and this association is not explained by coerced patients being more unwell than non-coerced patients. Strategic drives to reduce or eliminate the use of coercive measures, and seclusion in particular, may therefore have financial as well as ethical and clinical benefits [31], the cost of inpatient treatment remaining the main driver of costs in mental health care [32]. Staff education and training with the aim of reducing the use of coercive measures in mental health care should continue and gain momentum [33], with the potential cost savings justifying the required investment.

## Conclusions

Of the three coercive measures, forced medication appears to be unique in its significant impact on patient disapproval of treatment. All coercive measures are associated with patients staying longer in hospital, and seclusion significantly so, and this association is not fully explained by coerced patients being more unwell at admission. Strategies to reduce or avoid the use of coercive measures, and forced medication in particular, should be a focus of future research.
